# Monitoring Training Effects in Athletes: A Multidimensional Framework for Decision-Making

**DOI:** 10.1007/s40279-026-02417-4

**Published:** 2026-03-13

**Authors:** André Rebelo, Chris Bishop, Robin T. Thorpe, Anthony N. Turner, Tim J. Gabbett

**Affiliations:** 1 CIDEFES, Research Center in Sport, Physical Education, and Exercise and Health, Lusófona University, Lisbon, Portugal; 2 Faculty of Sport, Centre for Research, Education, Innovation, and Intervention in Sport (CIFI2D), University of Porto, Rua Dr. Plácido Costa, 91, 4200-450 Porto, Portugal; 3 COD, Center of Sports Optimization, Sporting Clube de Portugal, Lisbon, Portugal; 4 Faculty of Science and Technology, London Sport Institute, Middlesex University, London, UK; 5 Red Bull Performance Centre, 2700 Pennsylvania Ave, Santa Monica, CA 90404 USA; 6 Gabbett Performance Solutions, Brisbane, QLD 4011 Australia; 7 Integrative Human Performance Lab, College of Health Solutions, Arizona State University, Phoenix, AZ USA; 8 Department of Sport and Exercise Sciences, Institute of Sport, Manchester Metropolitan University, Manchester, UK

## Abstract

Athlete monitoring is widely used to support training and recovery decisions in elite sport, yet practitioners often face challenges related to data quality, feasibility, and the interpretation of short-term readiness signals within longer-term training adaptation. This narrative review synthesizes conceptual and applied developments in athlete monitoring through the lens of ‘training effects’, encompassing positive adaptation, maintenance, or maladaptation arising from training, competition, and contextual stressors. We distinguish assessment as isolated or periodic measurement from monitoring as repeated, systematic data collection used to track change over time. Building on contemporary conceptual models, readiness is positioned as an operational proxy for training effects that can inform day-to-day decision making when interpreted longitudinally and within context. We integrate the Minimal, Adequate, and Accurate framework to support tool selection that is economical in resource use, sufficient to meet clearly defined objectives, and grounded in valid and reliable measurement. Tools and metrics are organized according to the primary construct they inform: training load, athlete state and training response. We summarize practical considerations across neuromuscular, subjective, physiological, biochemical, and sleep-related indicators, emphasizing interpretive scope, measurement variability, and implementation constraints. To operationalize individualized monitoring, we outline pragmatic approaches using athlete-specific baselines and distribution-based thresholds (e.g., standard deviation intervals, minimum detectable change), alongside decision-making considerations related to Type I and Type II errors. Overall, this framework aims to reconcile scientific rigor with real-world feasibility, supporting practitioner decision making while acknowledging that monitoring should function as a decision-support process rather than a stand-alone determinant of performance outcomes.

## Key Points

**Table:** 

Athlete monitoring is commonly used in elite sport to support short-term decisions using readiness and fatigue indicators; this review reframes these signals as operational proxies for interpreting training effects across time.	
A practical, multidimensional monitoring framework is presented that links commonly used measures to their primary construct (training load, athlete state or training response), emphasizing that selection should be minimal, adequate, and accurate for the intended decision.	
Implementation is supported with applied interpretation tools, including individualized thresholds (e.g., standard deviation-based bands and minimum detectable change) and simplified visualization (quadrant model) to improve the linkage between measurement and action.	
The review prioritizes individualized interpretation by accounting for within-athlete variability and measurement error, offering accessible statistical strategies to distinguish meaningful change from normal noise in day-to-day monitoring.	

## Introduction

The role of sport in society has evolved significantly over the past several decades, with many disciplines experiencing a shift from recreational or amateur participation to increasingly professionalized and commercialized models [1]. To meet increasing commercial and competitive demands, competition calendars across all levels of sport have expanded in both duration and density. Athletes in individual and team-based disciplines alike now face greater physical and psychological challenges, with frequent matches, tournaments or qualification cycles occurring throughout the year. This intensification of the competitive environment has placed new emphasis on training efficiency, recovery management, and data-driven decision making [2]. Regardless of the sport, one of the key strategies used to sustain high performance is the monitoring of athlete readiness, which allows support staff to interpret the effects of training and competition, and make timely, individualized adjustments as required [3].

Team sports, such as rugby, volleyball, basketball, handball and football, are globally popular and widely played at both amateur and professional levels. These sports share common characteristics, including being fast paced, highly strategic, and requiring coordinated efforts from players towards a common goal (i.e., winning). At the elite and world-class levels, the competitive calendar for these sports is demanding [4]. As a result, they may face schedules with one or more matches per week over extended periods of time. This high frequency of matches, combined with the demands of training, travel and recovery, creates a unique challenge for managing athlete readiness and maintaining performance [5]. The nature of these schedules highlights the importance of effective monitoring and management strategies. With limited recovery time between matches and training sessions, athletes are at a heightened risk of fatigue and injury, making training-effects monitoring an important component of performance optimization.

In individual or self-paced sports such as athletics, swimming, cycling or combat sports, athletes face a distinct but equally complex set of demands. While they may not contend with the same tactical variability or interpersonal dynamics found in team environments, these athletes are often exposed to prolonged periods of high training volumes, intense performance pressure, and tightly packed qualification windows [6]. Success in these sports typically hinges on achieving peak performance at precisely scheduled moments (e.g., national trials, World Championships, or Olympic events), making the timing of training loads and recovery even more critical [6]. Furthermore, individual athletes often take on a greater share of responsibility for monitoring their own training effects, particularly in decentralized training models where daily supervision by coaches or support staff may be be limited [7]. This autonomy demands that monitoring strategies be both user-friendly and meaningful, enabling athletes to self-regulate effectively without relying solely on subjective judgement. In these sports, training load and recovery demands are often high, and monitoring can support early identification of excessive fatigue and insufficient recovery, which are associated with maladaptive outcomes [7].

Training effects refers to the acute and chronic responses athletes experience as a result of exposure to training, competition and recovery, encompassing positive adaptations, maintenance of performance capacity, or negative outcomes such as maladaptation and injury risk [8]. Athlete readiness, a related but narrower construct, describes the athlete's immediate state of preparedness to train or compete effectively, both physically and mentally, at a specific point in time [9]. Within a multidimensional framework, readiness acts as a practical indicator reflecting underlying training effects rather than being an isolated concept per se. In this context, it is important to distinguish between assessment and monitoring. Assessment typically refers to isolated or periodic measurements used to characterize an athlete’s status at a given point in time, whereas monitoring involves the repeated and systematic collection of such measures to track changes over time and inform training and recovery decisions. A broad approach to assessing training effects and readiness typically includes monitoring muscular strength, endurance, cardiovascular markers such as heart rate variability (HRV), and cognitive function [8, 10]. Importantly, this assessment extends beyond purely physical indicators to often include health-related factors such as sleep quality and subjective evaluations of muscle soreness, stress levels and mood [11]. This holistic perspective highlights the interconnectedness of physical, physiological and psychological dimensions, recognizing that optimal performance depends on an athlete’s overall well-being [12]. The value of monitoring training effects through readiness metrics lies in providing teams and coaches with actionable insights into an athlete’s evolving status. By identifying periods of reduced readiness or emerging maladaptation, coaches can adjust training and recovery strategies to minimize injury risk, reduce overtraining, and maximize performance potential [3, 7, 13]. Regular monitoring of training effects and readiness indicators equips coaching staff with the tools to make informed decisions about workload management, ultimately supporting athletes in reaching their peak capabilities.

Monitoring training effects and athlete readiness presents a variety of challenges for coaches and practitioners, stemming from the complexity of gathering, interpreting and acting on the data. In the context of elite sports, these challenges are amplified by the dynamic and multifaceted nature of training effects, influenced by both internal and external stressors. One major challenge is the reliance on data-driven monitoring, which, while promising, often introduces significant obstacles [14]. The pressure to adopt advanced technologies, such as wearables and software platforms, can lead to the indiscriminate use of tools and algorithms that lack proper validation [15]. When metrics are selected without a clear understanding of their validity or reliability, the risk of generating inaccurate or misleading data increases. Poor-quality data not only hinders effective decision making but also results in unnecessary costs and inefficiencies (e.g., time wasted interpreting noisy or irrelevant data, financial investment in tools that do not influence training decisions, or athlete frustration due to excessive testing). To address this, a data-driven approach is needed, where only metrics with appropriate measurement precision are prioritized to answer specific, actionable questions [14]. This perspective aligns closely with the ‘Minimal, Adequate, and Accurate’ (MAA) framework proposed by Washif and Pyne [16], which advocates for the selection of monitoring tools that are economical in resource use (minimal), sufficient to meet clearly defined objectives (adequate), and grounded in valid and reliable measurement (accurate). The MAA framework emphasizes that monitoring systems should prioritize usability, sustainability and measurement quality, ensuring that collected data meaningfully inform training decisions without overburdening athletes or staff [16].

The balance between objective and subjective measures is another important consideration. Objective metrics, such as force output or HRV, provide quantifiable insights but often fail to capture the holistic and adaptive nature of an athlete’s state [17]. Conversely, subjective measures, such as perceived fatigue or mood, offer a more integrated understanding of performer-environment interactions but may be prone to bias and deceit [17]. Evidence suggests that combining both objective and subjective measures offers the most comprehensive approach, enabling coaches to contextualize data and make more informed decisions [18]. Finally, the highly contextual and sport-specific nature of athletic performance adds another layer of complexity to monitoring training effects. While physical capacities are important across all disciplines, the degree to which performance is influenced by tactical, psychological and environmental factors may vary depending on the sport. For example, in team-based environments, additional considerations such as coordination with teammates, game strategy, and role-specific demands can impact athletes’ responses to training stimuli [19]. Individualizing monitoring approaches in any setting requires integrating multiple dimensions, including training load, recovery strategies, and nutrition, while remaining sensitive to the context and structure of the sport in question [5].

While athlete monitoring is widely used to inform training and recovery decisions, direct experimental evidence demonstrating superior performance outcomes resulting solely from monitoring-informed training is limited; therefore, monitoring should be viewed primarily as a decision-support process that aids practitioners in interpreting training responses, managing fatigue, and individualizing training loads. With all this in mind, this article aims to address these challenges by providing a narrative review of the concept of training effects across a variety of sports. The primary objective is to offer practical applications that practitioners can use to monitor training effects through readiness indicators on a daily basis, enabling more informed decisions about training and recovery. Furthermore, this article proposes a framework for monitoring training effects that integrates both objective and subjective measures, offering a practical and holistic guide to optimize performance, manage fatigue, and reduce injury risk in high-performance environments. Importantly, this review also acknowledges the ongoing tension between scientific rigor and real-world feasibility [16, 20], aiming to highlight tools and strategies that provide appropriate measurement precision, while remaining accessible, sustainable and applicable in daily practice. As a narrative review, this article does not follow a formal systematic search strategy. Instead, the literature was identified through an iterative and targeted approach, drawing on the authors’ expertise in athlete monitoring, training load, and performance science. Peer-reviewed articles, consensus statements and key reviews were identified through focused searches of major scientific databases and by examining reference lists of relevant publications.

## Conceptual Developments in Athlete Monitoring

### Evolution from Fatigue-Based to Training Effects-Based Monitoring

For many years, athlete monitoring has largely focused on detecting states of fatigue, under-recovery or ‘readiness’ to train and compete [21]. These binary classifications (ready vs. not ready) have formed the basis for decisions around load management, recovery interventions and injury prevention. However, a growing body of literature now challenges the idea that short-term readiness alone should drive decision making. Jeffries et al. [8] argue that this narrow focus may limit practitioners’ ability to understand the full spectrum of responses to training. A conceptual framework, in this context, refers to an interpretive model that helps organize and make sense of complex phenomena (i.e., the physiological and psychological effects of training), by offering a structured way to understand how different components relate to each other. Instead, Jeffries et al. [8] propose a shift toward interpreting data as reflections of training effects, defined as the cumulative outcomes of all sport-related demands, including physical preparation, competition and contextual stressors, that may be either positive (adaptation), neutral (maintenance) or negative (maladaptation). This shift moves away from labelling all deviations from baseline as inherently negative and calls for a more nuanced understanding of performance responses and adaptations in the context of long-term athlete development.

For example, a transient reduction in neuromuscular performance or subjective wellness may be expected, or even desirable, during a high-load training block designed to induce overload, provided it aligns with the intended adaptation phase and the athlete is coping well overall [22]. Similarly, consistently stable or ‘neutral’ metrics may not always be desirable if the goal is to stimulate adaptation through overload. In this framework, fatigue is no longer treated as a problem to be avoided at all costs, but one of several training effects that must be evaluated within a broader performance and recovery context [8]. By reframing monitoring data in this way, practitioners can better distinguish between acceptable and expected responses, and those that require intervention.

This evolution reflects an important development in how monitoring is used in applied sport settings. Rather than relying solely on reactive decisions triggered by single day deviations, the training-effects model promotes a context-sensitive and process-driven approach [8]. It aligns closely with contemporary views of periodization, athlete development, and individualized load management, where short-term fluctuations in readiness indicators are interpreted within the trajectory of long-term goals and monitored trends [23–25]. In this sense, readiness should not be discarded but instead used explicitly as one of several practical indicators within a larger interpretive framework that values long-term adaptation over short-term variability [26]. This shift encourages practitioners to consider not just whether the athlete is ready today, but whether they are adapting as intended over time.

### Defining What We Monitor: Fatigue, Adaptation and Readiness

**Fig. 1:**
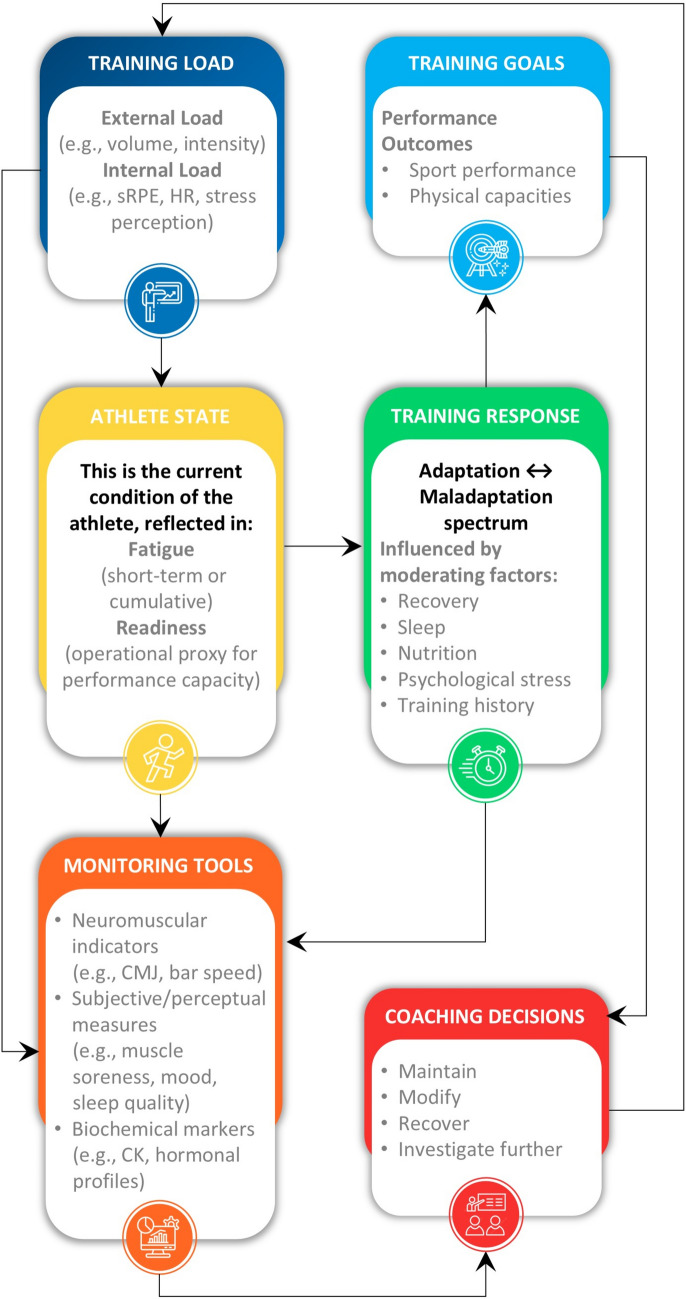
Conceptual overview of the fatigue–readiness–adaptation continuum in athlete monitoring. This diagram illustrates the dynamic relationship between training input, physiological and psychological response, short- and long-term fatigue, readiness fluctuations, and potential adaptation outcomes. It highlights how internal and external load stimuli lead to a cascade of responses that can result in performance impairment, stability or positive adaptation, depending on how stress is managed, and recovery is integrated

Before selecting tools, metrics or frameworks, it is important to establish what athlete monitoring is fundamentally trying to observe. To ground the discussion that follows in this paper, we first define the key constructs of fatigue, training response and readiness, and clarify the role each plays in the purpose of monitoring—a framework illustrated in Fig. 1. To promote clarity and consistency throughout this article, key monitoring concepts and their operational definitions are summarized in Table 1.

**Table 1:** Operational definitions of key monitoring concepts

Term	Operational definition	Primary use in monitoring
Fatigue	A reversible reduction in an athlete’s physical or psychological performance capacity due to training, competition, or life stress [7, 27]	To identify accumulated stress and adjust training loads accordingly
Acute fatigue	Immediate reductions in performance capacity within or shortly after a single training session or competition bout, typically resolving within minutes to hours [28]	To inform between-session, with-in-day decisions and post-session recovery strategies
Residual fatigue	Lingering fatigue from previous days’ training or competitions, typically persisting for 1–2 days [28]	To monitor between day recovery and inform short-term training decisions
Cumulative fatigue	Fatigue that builds and is added to, over several sessions due to sustained training or life stress. It is not inherently negative and may be a necessary part of overload and adaptation, provided it is well managed and followed by adequate recovery [29]	To evaluate training stress accumulation and support load management across micro- or mesocycles
Long-term fatigue	A maladaptive fatigue state resulting from prolonged imbalance between training load and recovery, potentially leading to performance decrements or injury [29]	To detect chronic maladaptation and guide long-term load management and recovery strategies
Readiness	The athlete’s immediate state of preparedness to train or compete effectively, both physically and mentally, at a specific point in time [7, 8, 17]	For real-time or daily decision-making regarding training adjustments or recovery
Training response	The cumulative physiological and psychological adaptations (positive or negative) that occur in response to training and life stressors over time [27]	To assess longer-term outcomes such as adaptation, maintenance, or maladaptation
Training effects	A conceptual framework that integrates fatigue, readiness, and adaptation to understand how athletes respond to imposed demands [8]	To guide overall programming and interpret monitoring data over time

Fatigue can be broadly understood as a temporary and reversible reduction in an athlete’s capacity to perform [7, 27]. It is not a singular construct but rather a complex interaction of physiological, neuromuscular and psychological factors that manifests over varying time courses. For greater clarity, fatigue can be conceptualized as occurring in four main forms. Acute fatigue arises immediately during or after exercise, such as between sets or at the end of a training session. Residual fatigue lingers from previous sessions, typically from the day before or earlier in the week, and may resolve within 24–48 h [28]. Cumulative fatigue develops over longer training blocks due to sustained demands, requiring more prolonged recovery to resolve. Finally, long-term fatigue reflects a chronic state often linked to over-reaching or overtraining, where performance declines persist and may be accompanied by mood disturbances, hormonal disruption or illness [29]. Fatigue can also be classified by its origin within the nervous system: central and peripheral. Central fatigue is associated with reduced voluntary activation of muscle, caused by decreased motor cortex drive or impaired motoneuron firing [30]. In contrast, peripheral fatigue originates within the muscles themselves, typically involving impaired contractile function and disrupted muscle action potential transmission [30]. From an applied perspective, the relative contribution of central and peripheral fatigue is strongly influenced by the nature of the training stimulus. Prolonged endurance exercise, high training volumes, sleep restriction, psychological stress and cognitive demands have been associated with greater central fatigue, mediated through altered motor drive, neurotransmitter balance, and perceptual regulation of effort [30]. In contrast, activities characterized by high mechanical loading, repeated eccentric actions, maximal or near-maximal force production, sprinting and change-of-direction tasks are more likely to induce peripheral fatigue, driven by metabolite accumulation, excitation–contraction coupling impairment, and muscle damage [30]. While often viewed negatively, fatigue is not inherently detrimental. As highlighted in Gabbett’s training-performance/injury model [31], periods of well-managed fatigue are necessary for adaptation. The key concern is not the presence of fatigue itself, but whether fatigue accumulates faster than the athlete can recover or adapt, which may increase injury risk or impair performance.

Training response refers to the body’s adaptation to the total training load over time, encompassing both the intended physiological improvements (e.g., aerobic fitness, strength, neuromuscular coordination) and unintended maladaptations (e.g., overreaching, illness, injury) [27]. As described in two International Olympic Committee consensus statements [2, 32], training effects are context-specific and depends not only on training load but also on an athlete’s capacity to tolerate and recover from that load. This capacity is shaped by factors such as training history, sleep, nutrition and psychosocial stress [2, 32]. Importantly, training response and adaptation are not directly observable at a single time point; instead, they emerge progressively across days or weeks and must be inferred from longitudinal patterns in performance, physiological and perceptual indicators.

In contrast, readiness refers to an athlete’s current functional capacity to train or compete and represents a short-term, time-specific expression of their underlying training state [7, 17]. While readiness does not explain training effects comprehensively, it serves as a proxy, offering coaches a real-time inference about the athlete’s ability to perform or train effectively at a given moment [8]. Its primary use is to aid short-term decision-making (i.e., whether to adjust today’s training, intervene with recovery strategies, or investigate deeper patterns). As Halson [7] emphasizes, readiness tools should not be judged solely by their ability to predict performance or injury, but by their role in guiding day-to-day decisions.

Therefore, the purpose of monitoring is not limited to detecting fatigue, nor is it solely about measuring readiness. It is about using a combination of tools to navigate the relationship between training load and training response, with the goal of supporting adaptation while minimizing maladaptation [2]. Monitoring systems should help identify whether an athlete is progressing as planned, showing early signs of maladaptation, or experiencing a disconnect between imposed load and response [2, 27]. Within a team sport environment, it is a tool to individualize group training. In this view, readiness becomes the operational tool, while training effect is the conceptual goal. Finally, it is worth acknowledging that even widely used terms like ‘short-term fatigue’ or ‘acute readiness’ often lack precise definitions in the applied literature [8]. By explicitly framing these constructs and their interrelationships, practitioners can improve the clarity and consistency of their monitoring systems. More importantly, they can better align their monitoring practices with the dual goals of enhancing performance and reducing injury risk. As such, we advise the terms and associated definitions as highlighted in Table 1.

### Readiness as a Window into Training Effects

While the shift toward understanding training effects offers a more explanatory model of athlete monitoring, it remains challenging to apply directly in many high-performance environments [33]. In contrast, readiness is inferred from multiple indicators, including markers of fatigue, recovery and neuromuscular function, which together reflect the athlete’s current functional state, and is more accessible and interpretable for coaches and support staff in day-to-day settings [17, 33]. These measures offer valuable, if indirect, insights into how athletes are responding to training. When interpreted consistently over time using contextual benchmarks and individual baselines, they provide a practical approximation of the athlete’s position within a broader continuum of training effects. For instance, a sustained decline in readiness metrics may signal an emerging maladaptive response to training, such as unresolved or poorly managed accumulated fatigue or insufficient recovery, which align with the maladaptation category within the training-effects model. Conversely, consistent improvements in neuromuscular performance or subjective well-being can be interpreted as markers of positive adaptation. Flat or stagnant values, especially when training load has not been appropriately manipulated, may reflect a no-adaptation scenario. However, in a congested competitive period, such values may also reflect effective short-term recovery interventions, maintaining function despite external stressors. In this sense, readiness becomes a measure that, once interpreted alongside the athlete’s acute training and life history, can shape the coach’s reflective practice and guide immediate and subsequent decisions. In this sense, readiness becomes a practical indicator that, when interpreted longitudinally and within context, can inform how athletes are tolerating imposed training demands.

### Practical Constraints in the Application of Training-Effects Models

Although the theoretical progression towards interpreting data through the lens of training effects provides a more comprehensive model for athlete development, its implementation in real-world environments remains constrained by several practical limitations. Most notably, high-performance settings often demand timely, actionable insights that can be easily communicated across multidisciplinary teams [10, 17]. Unlike conceptual models that involve detailed physiological reasoning or longitudinal analysis of training adaptations, applied environments sometimes rely on simplified tools to manage large groups of athletes with limited resources. This disparity between conceptual depth and operational feasibility creates a tension between academic and applied sport scientists: while the former seek mechanistic understanding, the latter prioritize real-world decision making under time constraints. Within this context, readiness indicators—though limited—serve as practical proxies to inform immediate adjustments. This pragmatic reliance on streamlined indicators aligns with the MAA framework proposed by Washif and Pyne [16], which advocates prioritizing the smallest set of valid and reliable measures required to generate decision-relevant information under real-world constraints. Within this framework, readiness indicators function as minimal inputs that are adequate for guiding day-to-day decisions, provided they meet standards of measurement accuracy.

One of the primary challenges lies in the volume and complexity of data required to meaningfully evaluate training effects [34]. Mechanistic interpretations often demand integrated analyses of multiple physiological systems, including hormonal profiles, neuromuscular outputs, subjective well-being and performance outcomes, collected over extended periods. However, in daily practice, practitioners frequently lack the time, budget or staff to collect, process and interpret such comprehensive datasets. In many cases, coaches must make decisions within minutes, not days, and must base these on brief morning assessments, training observations, discussions with the athlete, or automated software summaries. As a result, while models based on training effects offer conceptual clarity, they are often inaccessible or underutilized due to the constraints of the environment. This reinforces the value of simplified readiness assessments as operational tools, particularly when aligned with individual baselines and interpreted within context.

Additionally, athlete buy-in and adherence play a critical role in monitoring success [17]. Tools that are perceived as overly invasive, time-consuming or difficult to interpret may face resistance from players or staff, regardless of their scientific rigor [17, 35]. In contrast, more intuitive, lower-burden assessments, such as wellness questionnaires or CMJ scores, tend to be better accepted and more consistently implemented, even if they offer a narrower window into the full complexity of training adaptations. While these measures offer limited insight into the mechanisms of adaptation, their primary role in practice is operational rather than explanatory, supporting short-term decision making under real-world constraints. Recent evidence from a scoping review of coaches’ and support staff’s perspectives indicates that monitoring is generally perceived as valuable for informing training, reducing injury and illness risk, and supporting performance, but its perceived effectiveness is often lower than expected due to practical barriers such as time, expertise, resources and challenges in obtaining athlete buy-in and compliance [36]. Importantly, coaches and support staff emphasized that monitoring should not replace regular communication and should be integrated as part of a broader decision-making process [36]. Lastly, it is important to recognize that not all performance environments are equipped with the same level of expertise, equipment or support infrastructure. While some elite organizations have access to sport scientists, performance analysts, and integrated teams capable of managing complex datasets, many teams operate with limited staff and minimal technological resources. In these settings, practitioners often rely on streamlined indicators, such as submaximal heart rate (HR) responses, subjective wellness ratings, or selected jump metrics, to infer short-term changes in athlete status. When grounded in individual baselines and clear interpretive frameworks, these simplified tools offer a scalable entry point into the broader monitoring of training effects, helping coaches navigate daily decisions without exceeding their operational capacity.

**Fig. 2:**
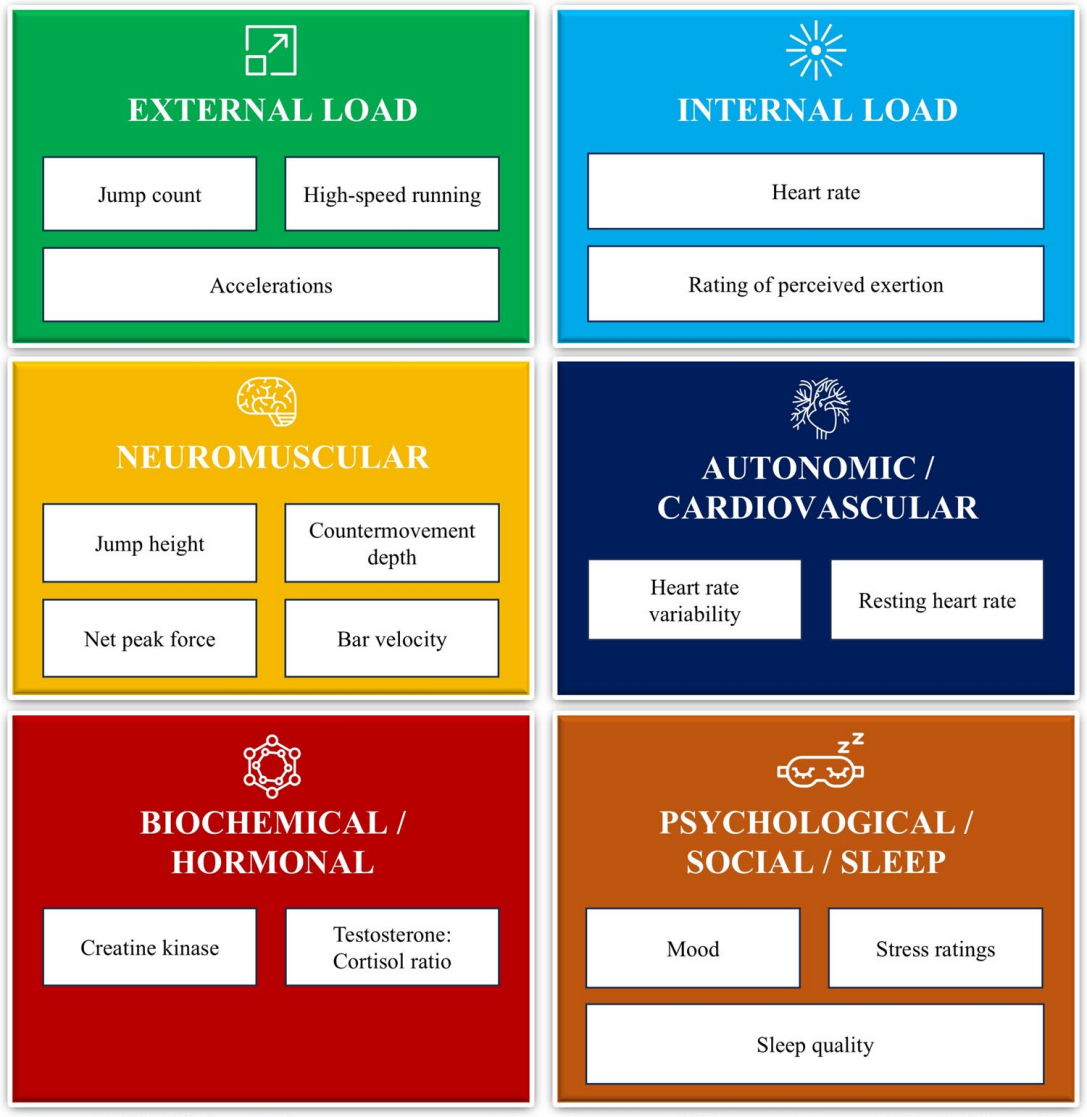
This figure presents key monitoring domains and example metrics used to assess training effects in high-performance sport. The model emphasizes a broad, integrated approach, encouraging practitioners to select context-appropriate measures from multiple physiological and psychological systems to construct a comprehensive athlete profile

By selectively integrating information across multiple monitoring domains, such as external and internal load, neuromuscular performance, autonomic and cardiovascular function, and psychological or social state, coaches can build a comprehensive and context-sensitive picture of an athlete’s current status (Fig. 2) [12]. Importantly, such integration does not imply that all domains must be monitored concurrently; rather, practitioners can combine a limited number of feasible indicators across domains to build a sufficiently informative and context-sensitive overview. This multidimensional framework supports more responsive and informed programming, helping practitioners navigate the balance between long-term training adaptations and short-term readiness for performance or recovery. However, while clustering measures can provide useful overall trends, practitioners should ensure that each metric directly reflects the physiological or psychological system of interest, allowing for clearer interpretation and targeted intervention. Ultimately, this paper embraces readiness as a practical entry point to monitor, interpret and respond to training effects. It offers a framework and selection of tools that, while not exhaustive or mechanistically complete, are tailored for real-world implementation. In doing so, it aims to bridge the gap between theoretical precision and operational practicality, supporting better-informed, athlete-centred decisions in high-performance sport environments.

## Monitoring Training Load, Athlete State and Training Response: Tools and Metrics

This section summarizes the tools and metrics commonly used to monitor training load, athlete state and training response in applied sport settings. In line with the conceptual framework presented in Fig. 1, tools are organized according to the primary construct they inform, acknowledging that some measures may contribute to multiple constructs depending on measurement timing and context. The focus is placed on practical applicability, measurement considerations, and interpretive scope rather than exhaustive methodological detail.

### Monitoring Training Load

Within the monitoring framework (Fig. 1), training load represents the imposed stimulus of training and competition and constitutes the primary input to the athlete monitoring system. Training load refers to the total amount of stress imposed on an athlete through structured physical activity and is a central concept in both performance development and injury prevention [37]. It is commonly divided into two main components: external load and internal load [38]. External load encompasses the objective work completed by the athlete, such as distance covered, weight lifted, speed, number of jumps, or accelerations performed [38]. These measures are typically derived from tracking devices, gym-based data, or training plans, and are specific to the type of activity performed. In contrast, internal load represents the immediate physiological and perceptual response of the athlete to that external work, commonly quantified using measures such as HR or session rating of perceived exertion (sRPE) [38]. While internal load reflects the acute cost of training, it should be distinguished from athlete state indicators (e.g., fatigue or readiness), which describe the athlete’s current functional status. Both internal and external loads provide complementary insights: the external load reflects the dose of training, while the internal load captures the individual response to that dose. Importantly, neither dimension has a universally accepted gold-standard metric. The selection of appropriate indicators depends heavily on the training context and sport demands. For example, HR may be highly valid for monitoring internal load during aerobic conditioning but less effective for short-duration, high-intensity efforts like sprinting or strength training. Accordingly, training load metrics are primarily used to describe what was prescribed and completed, providing important context for interpreting subsequent changes in athlete state and training response.

### Monitoring Athlete State

#### Neuromuscular and Performance-Based Indicators

Within the monitoring framework (Fig. 1), neuromuscular and performance-based indicators primarily inform the athlete state by reflecting short-term fatigue expression and readiness to train or compete. These tests are most informative when performed in close temporal proximity to training (e.g., pre-session or post-session) and interpreted relative to individual baselines.

Velocity-based training (VBT) offers a practical and objective approach to assess neuromuscular readiness without requiring maximal testing [39]. Because bar speed at a given load correlates with fatigue and performance capacity [40], tracking barbell velocity can help detect day-to-day fluctuations in readiness due to factors like sleep [41], nutrition [42], or psychological stress [43]. Mean propulsive velocity at moderate to heavy loads [40–80% 1RM (repetition maximum)] demonstrates similar sensitivity to fatigue as CMJ height, particularly at 40% and 80% 1RM where post-exercise changes showed moderate agreement (ICC = 0.71 and 0.58, respectively; CV = 5.1–8.5%) [44]. Additionally, velocity loss thresholds can be applied to regulate training volume and intensity. When bar speed drops beyond a set threshold, practitioners can adjust training in real time to prevent excessive fatigue [39]. Linear position transducers are among the most usable devices for measuring bar velocity, with high validity (*r* = 0.96–0.98) and acceptable reliability (ICC ≈ 0.67; CV = 6.6%) [45]. However, newer, low-cost options (e.g., smartphone apps) are emerging [46]. While effective, these metrics are not immune to external factors like motivation or technique variability and should be interpreted in conjunction with contextual indicators (e.g., perceived exertion) [39].

Isometric strength testing, involving muscle contractions without joint movement, offers an alternative for assessing neuromuscular readiness, particularly when maximal dynamic efforts are not feasible [47]. These assessments are useful in both rehabilitation and performance settings due to their safety, simplicity and objectivity. Among the most reliable indicators is net peak force, which has demonstrated high test–retest reliability (ICC ≥ 0.80, often up to 0.99) [48], making it suitable for tracking fatigue or performance impairments. However, implementation challenges exist, especially in large teams. Standardizing joint angles (e.g., knee, hip) across athletes requires time, equipment and trained personnel, reducing feasibility in time-constrained environments. Additionally, while rate of force development is sometimes used, it is more sensitive to testing inconsistencies and thus less reliable [49]. Practitioners are encouraged to prioritize robust metrics, such as peak force, when integrating isometric testing into readiness monitoring protocols.

The CMJ is one of the most widely used tests for monitoring short-term neuromuscular fatigue and pre-session performance readiness in elite athletes [50] due to its simplicity [51], efficiency [52], and non-invasive nature [51]. While jump height and peak power are traditionally used [50], they may lack sensitivity to short-term fatigue, as athletes can maintain jump height by altering their jump strategy [53]. Instead, time-based and strategy-related variables, such as time to take-off and countermovement depth, offer greater sensitivity to fatigue-related changes [53]. For instance, after intensive training, both metrics have shown large reductions (effect sizes ≥ 0.8) with CV values typically < 10%, indicating acceptable reliability [54]. These shifts reflect subtle neuromuscular impairments, as fatigued athletes often prolong the force application phase to compensate for reduced output. In terms of feasibility, CMJ testing, especially using force plates, is time-efficient, easily integrated into warm-ups, and scalable to large teams. Portable systems and user-friendly software further enhance its accessibility, although cost and expertise may limit use in some settings [54].

Short-duration cycle ergometer tests, such as the peak power protocol on a Wattbike, offer a reliable, non-weight-bearing alternative for assessing neuromuscular fatigue in team sport athletes [55, 56]. The protocol, consisting of two 6-s sprints and minimal warm-up, requires just 2–3 min per athlete, making it highly feasible for moderate to large groups. However, the limited warm-up may increase variability in performance and elevate the risk of Type I errors, particularly if the warm-up is not standardized across sessions. To improve repeatability, practitioners are advised to implement a consistent short warm-up protocol before testing. It can be administered by a single staff member with minimal disruption, and its reliability is excellent (ICC = 0.96, CV = 3.0%) [56].

#### Subjective Indicators of Fatigue and Readiness

Psychological and social factors significantly influence athlete readiness, especially in high-pressure environments where personal, competitive and interpersonal demands intersect. Monitoring these domains offers a low-cost and scalable means to detect accumulating stress, motivation changes, or early signs of burnout. Validated tools like the Recovery-Stress Questionnaire for Athletes (RESTQ-Sport) [57] and the Daily Analysis of Life Demands for Athletes (DALDA) [58] help quantify emotional states and identify stress sources across sport and everyday life. These tools support proactive management of readiness by enabling early intervention when athletes report increasing strain, irritability, or reduced recovery. Their feasibility, requiring less than 5 min and no equipment, makes them well suited for regular monitoring in elite and team settings. However, interpretation should emphasize trends over time and consider subscale variability [59]. Composite factor scores, such as overall stress or recovery, tend to show greater reliability than individual items [60]. Ultimately, fostering a culture of trust is essential for accurate self-reporting and effective psychological monitoring. Athletes must feel confident that their responses will be used to support their well-being and performance, rather than being viewed as a reason for reduced playing time or exclusion from selection.

Athletes frequently experience physical discomfort that may not be detectable through objective assessments, making subjective self-reports a valuable tool for monitoring readiness [59]. Commonly tracked sensations include muscle soreness, pain and fatigue, which directly affect an athlete’s ability to train, compete and recover [61]. Muscle soreness, a natural response to physical exertion, can indicate either adaptive training responses or incomplete recovery depending on its intensity and duration [62]. Self-reported scales help identify patterns of excessive soreness, supporting tailored recovery protocols. Similarly, perceived fatigue, often assessed through brief questionnaires like the Hooper Index, can highlight accumulated load and insufficient recovery [63]. Pain requires more nuanced interpretation, as it may stem from structural injury or non-structural sources such as stress or fear of movement. The Oslo Sports Trauma Research Center (OSTRC) Questionnaire enables athletes to report pain and its impact on participation, helping staff respond appropriately through medical evaluation or training modifications [64]. These self-reported tools are highly feasible (although their utility ultimately depends on the quality and honesty of athlete responses), requiring minimal time (< 1 min), no equipment, and easy integration into digital platforms. Their low cost and scalability make them ideal for large squads or low-resource environments. While test–retest reliability varies, consistent implementation and individual baseline tracking enhance their value in detecting meaningful trends [65].

Despite their practical advantages, the effectiveness of subjective monitoring tools is highly contingent on data quality and athlete engagement. Recent qualitative research has shown that self-reported data may be intentionally or unintentionally distorted when athletes perceive a lack of trust, unclear purpose or potential negative consequences associated with their responses [66, 67]. Coventry et al. [66] reported that elite athletes may under-report fatigue or soreness when they fear repercussions related to selection, training modification, or staff judgement. Similarly, McCall et al. [67] demonstrated that athletes’ willingness to report honestly is strongly influenced by interpersonal factors, including transparency, perceived empathy, and the extent to which feedback leads to visible and meaningful action. These findings highlight that subjective monitoring should not be viewed solely as a measurement issue, but as a socially mediated process embedded within coach–athlete and practitioner–athlete relationships. When athletes understand why information is collected and observe consistent, supportive responses to their input, subjective measures can provide sensitive and ecologically valid insights into fatigue and readiness. Conversely, poor implementation may compromise data quality, limiting interpretability regardless of the tool’s psychometric properties.

#### Physiological Markers of Fatigue and Readiness

Physiological markers provide valuable insight into an athlete’s current functional state and are particularly useful for assessing short-term fatigue and readiness within the athlete state component of the monitoring framework (Fig. 1) [68]. These measures are most informative when collected at standardized time points close to training or competition and interpreted relative to individual baselines. Recent integrative frameworks have similarly emphasized the strategic use of physiological markers to monitor fatigue and recovery, advocating the selective combination of high-precision methods for research contexts with more feasible tools (e.g., HR, HRV and neuromuscular tests) for day-to-day practice [69].

Autonomic and cardiovascular markers are among the most commonly used physiological indicators of readiness [70]. Resting HR and HRV, particularly the natural logarithm of the root mean square of successive differences (Ln rMSSD), are typically collected in the morning or during sleep to capture parasympathetic activity while minimizing external confounders [70–72]. These measures are sensitive to acute stress and recovery fluctuations over the previous 12–24 h and are therefore well suited to informing day-to-day readiness decisions. However, their utility can be limited by substantial day-to-day variability, which necessitates careful standardization of measurement conditions and interpretation against individual reference values. Weekly rolling averages based on at least three to four measurements per week provide more stable insights into short-term trends and help distinguish true physiological changes from normal biological noise [73].

Beyond resting measures, submaximal exercise heart rate (HRex) recorded during standardized aerobic tests reflects the athlete’s acute physiological state and tolerance to training load. When assessed under consistent conditions, HRex can inform readiness to train and, in some contexts, indicate emerging fatigue. For example, reductions in 4-min HRex strongly correlate with improvements in Yo-Yo IR1 performance (*r* =  − 0.57) [74] and higher HRex has been linked to injury risk in underloaded athletes [75]. A systematic review reported HRex reliability as high (ICC = 0.88; TE = 1.6% HRmax) [76]. Given its low cost, practicality and acceptable reliability, HRex represents a feasible physiological marker for regular readiness monitoring in applied settings.

Biochemical markers of muscle damage, particularly creatine kinase (CK), can also contribute to the assessment of fatigue and recovery status when interpreted within an appropriate temporal window. CK primarily reflects cumulative muscle stress and recovery processes when measured 24–72 h following exercise and is therefore not well suited for same-day readiness decisions [77]. Instead, elevated CK values within this time frame may indicate unresolved muscle damage or incomplete recovery, particularly in sports characterized by repeated eccentric loading [78]. Due to large inter-individual variability (CV = 20%) [79], CK should be interpreted using individualized baselines rather than absolute thresholds, with deviations of approximately 1.5–2 standard deviations (SDs) from an athlete’s typical range often used to flag atypical responses [7]. While CK can support fatigue and recovery assessment, its moderate reliability (ICC = 0.49–0.64) and logistical demands limit its usefulness for frequent monitoring, especially in large team environments [80].

Taken together, autonomic, cardiovascular and selected biochemical markers primarily inform the athlete state by reflecting the current balance between fatigue and recovery. Their value lies in supporting short-term decisions related to training readiness, particularly when measurements are standardized, interpreted longitudinally, and integrated with subjective and performance-based indicators.

### Monitoring Training Response

In contrast to markers of athlete state, indicators of training response are used to evaluate how athletes adapt to training over longer time scales. These measures are less informative for daily decision making and instead support the identification of positive adaptation, stagnation or maladaptive responses when interpreted longitudinally across training cycles.

Markers of inflammation and immune function provide insight into systemic responses to prolonged or excessive training loads [81]. Cytokines such as interleukin-6 (IL-6), tumour necrosis factor-alpha (TNF-α), and interleukin-10 (IL-10), along with C-reactive protein, reflect inflammatory and immune processes associated with training stress and recovery [78]. Elevated concentrations of these markers often occur following periods of intensified training or congested competition schedules and may indicate an increased risk of illness or impaired recovery capacity [82]. However, due to their invasive nature, cost and limited feasibility for frequent sampling, these markers are best suited for periodic assessment or targeted investigations rather than routine monitoring. Importantly, they are often considered lagging indicators, meaning that physiological disruption may already be established by the time changes are detected.

Endocrine and metabolic markers further contribute to the assessment of longer-term training response and stress balance. Blood urea nitrogen reflects protein metabolism and overall training load exposure, with chronically elevated values potentially indicating excessive workload or insufficient recovery [78]. Similarly, the testosterone-to-cortisol (T:C) ratio is commonly used as an index of anabolic–catabolic balance [83]. Sustained reductions in this ratio over time may signal maladaptation or non-functional over-reaching, particularly during periods of high training volume or insufficient recovery [84]. As with other biochemical markers, endocrine measures should not be interpreted in isolation or based on single time points. Their primary value lies in longitudinal trend analysis, where persistent deviations from an athlete’s normal range can inform evaluations of training effectiveness and recovery adequacy.

While some markers discussed in the previous section, such as HRV or CK, may also contribute to the interpretation of training response when tracked over extended periods, their role within this context depends on measurement frequency and analytical time frame. When interpreted longitudinally, these markers can help distinguish between expected fatigue associated with planned overload and maladaptive responses that compromise adaptation. Overall, indicators of training response support the evaluation of whether imposed training loads are producing the intended adaptations or leading toward maladaptation. Although their practical application is often constrained by cost and feasibility, these measures provide valuable physiological context when used selectively and integrated with load data, performance trends, and athlete-reported outcomes.

### Sleep and Recovery-Related Processes

Within the monitoring framework presented in Fig. 1, sleep is not treated as a standalone outcome but as a central recovery-related process that modulates both athlete state (fatigue and readiness) and longer-term training response. Acute disturbances in sleep primarily influence next-day readiness and perceived fatigue, whereas chronic sleep restriction or consistently poor sleep quality may impair recovery capacity and contribute to maladaptive training responses over time. Accordingly, the interpretation of sleep metrics depends strongly on the timing and frequency of assessment. Sleep is a fundamental component of recovery, influencing cognitive function, physical restoration, immune health, and emotional regulation [85]. However, athletes often experience disrupted or insufficient sleep, particularly during congested schedules, travel or heightened psychological stress. Irregular training times, late-night matches, early sessions, and pre-competition anxiety can all impair sleep quality, while excessive screen time may delay sleep onset and reduce overall sleep efficiency [85]. To monitor sleep, both subjective and objective tools are used. Self-report methods, such as sleep diaries, the Pittsburgh Sleep Quality Index (PSQI) [86], or the Athlete Sleep Screening Questionnaire (ASSQ) [87], are highly feasible and low-cost. However, they are susceptible to recall bias and limited in detecting nuanced changes. In elite athletes, the PSQI has shown only poor-to-moderate reliability (ICC = 0.45–0.55) and a minimum detectable change (MDC) of 3 points [88]. Objective tools like actigraphy, which use wrist-worn accelerometers, provide more reliable and continuous assessments of sleep duration and movement patterns [89]. Although actigraphy cannot accurately differentiate sleep stages or detect all wake episodes [90], it is a practical tool for team sports, especially when combined with sleep diaries. In contrast, polysomnography offers high accuracy but poor feasibility due to cost and logistical demands [91]. Poor sleep patterns, such as short sleep duration, prolonged sleep latency, or frequent awakenings, can impair cognitive performance, increase fatigue, and reduce decision-making ability, all of which impact sport performance [85]. Monitoring short-term changes in sleep allows practitioners to identify declines in next-day readiness and increased fatigue, particularly when assessed in close temporal proximity to training or competition [91]. Given its importance across multiple recovery systems, sleep is arguably the most impactful, modifiable, and cost-effective recovery tool available to high-performance athletes. When interpreted contextually and tracked longitudinally, sleep data can guide individualized interventions that support short-term readiness and inform evaluations of longer-term training response and adaptation.

To enhance conceptual clarity and practical applicability, Table 2 summarizes the monitoring tools discussed in this section and maps each metric to its primary role within the monitoring framework (Fig. 1), namely training load, athlete state (fatigue and readiness), or training response. Where relevant, tools that may inform multiple constructs are identified, with interpretation contingent on measurement timing and longitudinal context. This structured overview is intended to support practitioners in selecting and interpreting monitoring tools in a manner consistent with the proposed framework.

**Table 2:** Summary of common athlete-monitoring tools: theoretical rationale, feasibility, and sensitivity

Tool/Test	Metric(s)	Primary monitoring construct	Biological basis	Feasibility	Sensitivity
Resting HRV	Ln rMSSD, HRVrest	Athlete state (fatigue/readiness)	Reflects autonomic nervous system status, capturing parasympathetic activity and readiness. Strong theoretical link with recovery status and fatigue	Moderate to high: Requires wearable or smartphone-compatible devices; can be measured at home; low cost; minimal staff; however, daily compliance and data quality are challenges in large squads	Moderate: Sensitive to short-term changes in fatigue and stress. Individual trends over time are more meaningful than single-day values. Day-to-day variability limits one-off interpretability
Submaximal HR	HR during 4-min submaximal effort (e.g., 30–15 test)	Athlete state (readiness) Training response (when tracked longitudinally)	Reflects aerobic fitness, training adaptation, and fatigue. Elevated HRex indicates insufficient adaptation or increased stress. Causal link supported by correlation with performance and injury risk	Moderate: Easy to integrate into fitness testing. Requires only a heart rate monitor and standardized test protocol. One coach/staff member can implement for large squads. Data review is immediate	High: ICC ≈ 0.88; TE = 1.6% HRmax. Sensitive to meaningful within-athlete changes. Strong correlation with Yo-Yo IR1 performance and useful for short-term and chronic adaptation tracking
Blood biomarker	Serum CK concentration	Athlete state (recovery/fatigue) Training response (chronic elevation)	Reflects muscle membrane disruption and cumulative muscular stress due to eccentric loading and high mechanical demand. Correlates with training load and delayed recovery	Low to moderate: Requires blood sampling and often lab analysis; low cost per test, but logistics and staffing are a barrier for frequent use. Reporting takes hours to 1–2 days. Less feasible for large squads or daily use	Moderate: Sensitive to cumulative fatigue but not immediate readiness. High inter-individual variability (ICC 0.49–0.64; CV ~ 20%) limits interpretability. Better used for trend analysis over time than isolated values
Hormonal profiling	Testosterone:Cortisol (T:C) ratio	Training response (adaptation/maladaptation)	Reflects the balance between anabolic (testosterone) and catabolic (cortisol) activity. A declining T:C ratio over time is associated with overreaching, overtraining, or insufficient recovery. Best used for trend analysis	Low: Requires blood or saliva samples. Saliva sampling is less invasive and more practical, but has higher biological and technical variability. Lab analysis or validated ELISA kits are needed. Time to results: 24–72 h depending on protocol and logistics	Moderate: Variability is high (critical difference: ~ 78–148% for salivary-T and salivary-C). Best interpreted longitudinally. Sensitive to acute changes in stress or recovery, but may not detect subtle changes within small windows unless strict sampling controls are used
Sleep questionnaires (e.g., PSQI)	Sleep quality and duration	Athlete state (readiness) Training response (chronic sleep patterns)	Sleep is important for recovery and adaptation across cognitive, physical, and immune systems; poor subjective sleep is linked to performance declines and injury risk	Very high: free, quick to complete (< 2 min); easily distributed via apps or forms; ideal for large teams; no equipment and minimal staff time needed	Moderate: limited accuracy due to recall bias and subjective interpretation; PSQI ICC = 0.45–0.55; MDC = 3 points
RESTQ-Sport	Stress, recovery	Athlete state (fatigue/readiness) Training response (chronic stress)	Chronic psychological stress influences hormonal balance, sleep, motivation, and recovery capacity; self-report allows early detection of fatigue or burnout risk	High: no equipment; < 5 min with shortened forms; original version may be burdensome in large squads or frequent testing. Shortened versions were developed for improved compliance	Moderate to high: specially when tracking global factor scores over time; composite reliability *ρ* > 0.84; subscale reliability varies
Barbell velocity monitoring	Mean propulsive velocity, velocity loss	Athlete state (neuromuscular readiness/fatigue)	MPV reflects neuromuscular performance under load; velocity loss correlates with fatigue-induced decrements in force output and CMJ height	Moderate to high: Linear position transducers are valid and reliable; newer apps are cheaper but may vary. Quick to implement, but requires equipment and athlete buy-in	High: MPV and velocity loss are sensitive to neuromuscular fatigue. ICC ≈ 0.67; CV ≈ 6.6% Can inform daily adjustments when interpreted with context
Isometric strength testing	Net peak force	Athlete state (neuromuscular readiness/fatigue)	Captures the maximum force-generating capacity of a muscle group under controlled conditions and reflects neuromuscular status	Moderate: High accuracy but requires standardized joint angles and equipment. Less feasible in large teams unless multiple stations/staff are available	Very high: ICC ≥ 0.80, often > 0.90). Sensitive to fatigue and strength impairments. RFD is less reliable and not recommended as primary metric
CMJ	CM depth; mean braking force; TTTO; jump height	Athlete state (neuromuscular readiness/fatigue)	Reflect fatigue-related neuromuscular strategy changes; fatigued athletes prolong force application to maintain performance	High: Quick to administer, even in large teams. Portable force plates with intuitive software increase feasibility. Cost/expertise can limit access	High: Metrics show large effect sizes (≥ 0.8) and CVs < 10% following fatigue. More sensitive than jump height for detecting neuromuscular changes
Cycle ergometer (Wattbike)	Peak power	Athlete state (neuromuscular fatigue)	Reflects neuromuscular fatigue via reductions in high-intensity power output. Non-weight-bearing, allowing safe fatigue detection	High: ~ 2–3 min including warm-up. Easy to standardize. Requires only one staff member. Feasible even in team settings	High: ICC = 0.96; CV = 3.0%. Sensitive to within-athlete neuromuscular changes

*CK* creatine kinase, *CM* countermovement, *CMJ* countermovement jump, *CV* coefficient of variation, *HR* heart rate, *HRex* exercise-related heart rate responses, *HRV* heart rate variability, *HRVrest* resting heart rate variability, *ICC* intraclass correlation coefficient, *Ln rMSSD* natural logarithm of the root mean square of successive differences, *MDC* minimal detectable change, *MPV* mean propulsive velocity, *PSQI* Pittsburgh Sleep Quality Index, *RESTQ-Sport* Recovery-Stress Questionnaire for Athletes, *RFD* rate of force development, *T:C* the ratio between testosterone and cortisol, *TTTO* time to take-off

## Applying Monitoring Data to Support Training Decisions

Effective athlete monitoring has progressively shifted from generalized, group-based assessment toward more individualized interpretation strategies [92]. While group-level analyses are important for establishing the validity and reliability of monitoring tools, they often lack the resolution required to guide day-to-day decisions for individual athletes in high-performance environments [92]. As a result, practitioners are encouraged to complement group-derived reference values (e.g., smallest worthwhile change (SWC) or normative thresholds) with individualized baselines that account for each athlete’s typical variability and training context [10].

The primary aim of individualized monitoring is to identify changes that are practically meaningful rather than statistically significant alone. Simple distribution-based approaches, such as interpreting changes relative to an athlete’s own SD, offer a robust and accessible method for this purpose [93]. Such approaches align with applied recommendations emphasizing clarity, speed and interpretability in decision making, particularly in environments where complex statistical models may hinder rather than support practice [20, 94]. Accordingly, effective monitoring systems should prioritize methods that balance measurement reliability with timely, context-sensitive interpretation to support informed training decisions.

### Operationalizing Individual Monitoring in Applied Settings

While advanced statistical methods can provide detailed insights into athlete-monitoring data, their practical application in high-performance environments is often limited by time, expertise and resource constraints. Accordingly, applied monitoring requires operational approaches that balance statistical rigor with interpretability and feasibility.

To support this, practitioners should first establish individual reference values for each athlete, clearly defined performance or status markers derived from standardized testing protocols. These reference values serve as the contextual benchmark for interpreting day-to-day or week-to-week changes. As noted in the beginning of this section, such individualized baselines should ideally be complemented by well-powered group-level test–retest studies to ensure the methodological robustness of the tools being used. The SD is one of the most accessible statistical tools for this purpose. Ideally, it should be calculated from repeated test–retest assessments across multiple days under consistent conditions. This approach captures both measurement error and short-term biological fluctuations, aligning more closely with the type of changes practitioners aim to detect when monitoring readiness. While within-session SDs (from multiple trials in a single session) may offer insights into measurement consistency, they are not an adequate substitute when the goal is to flag meaningful daily variation. Therefore, wherever feasible, readiness-related thresholds should be based on test–retest variability over timeframes that reflect the intended monitoring context. The SD effectively captures an athlete’s typical measurement variability, providing a direct and interpretable reference point for detecting meaningful change. It is important to distinguish this from SD calculated over a prolonged training period, which may include both random variation and true performance changes—thus limiting its utility for detecting small but meaningful shifts. Although a greater number of repeated sessions can improve statistical precision, this may not always be practical in applied environments. In real-world settings, if athletes are well familiarized with the testing procedures, fully engaged, and properly warmed up, it may be feasible to estimate meaningful within-athlete variability from as few as two reliable test sessions conducted on separate days under consistent conditions. Ideally, these sessions should be performed in the absence of intervening training, as this better isolates the athlete’s typical variability without the confounding influence of adaptation or fatigue. This approach balances the need for accurate interpretation with the time constraints and demands of high-performance sport.

Secondly, thresholds for meaningful change should be based directly on these test–retest SD values. A single SD interval (± 1 SD) offers a sensitive yet practical starting point for interpreting daily or weekly readiness fluctuations, highlighting small but potentially critical shifts without being excessively conservative. If greater confidence in detecting true performance changes is required, expanding to ± 1.64 SD (approximately 90% confidence), ± 2 SD (approximately 95% confidence) or ± 3 SD (approximately 99% confidence) intervals may be warranted, albeit at the cost of potentially overlooking smaller changes that could be practically meaningful [95]. Alternatively, when test–retest SD and reliability estimates are available, practitioners may calculate a MDC at a desired confidence level (e.g., 75% or 95%) to formally define the smallest change that can be interpreted as real, beyond measurement error [96]. From an applied standpoint, MDC values can be further complemented by anchor-based estimates such as the minimum practically important difference (MPID) or minimum clinically important difference (MCID) [97]. These thresholds reflect the smallest change in a metric that corresponds with a meaningful outcome, whether in performance, recovery or injury risk. For instance, Thorpe et al. [98] showed that a 1-unit decrease in perceived fatigue was associated with a ~ 400-m increase in high-speed running the day prior, suggesting that a change of that magnitude may be practically relevant and actionable. Such contextual benchmarks can enrich interpretation and support more confident decision-making in the field.

**Fig. 3:**
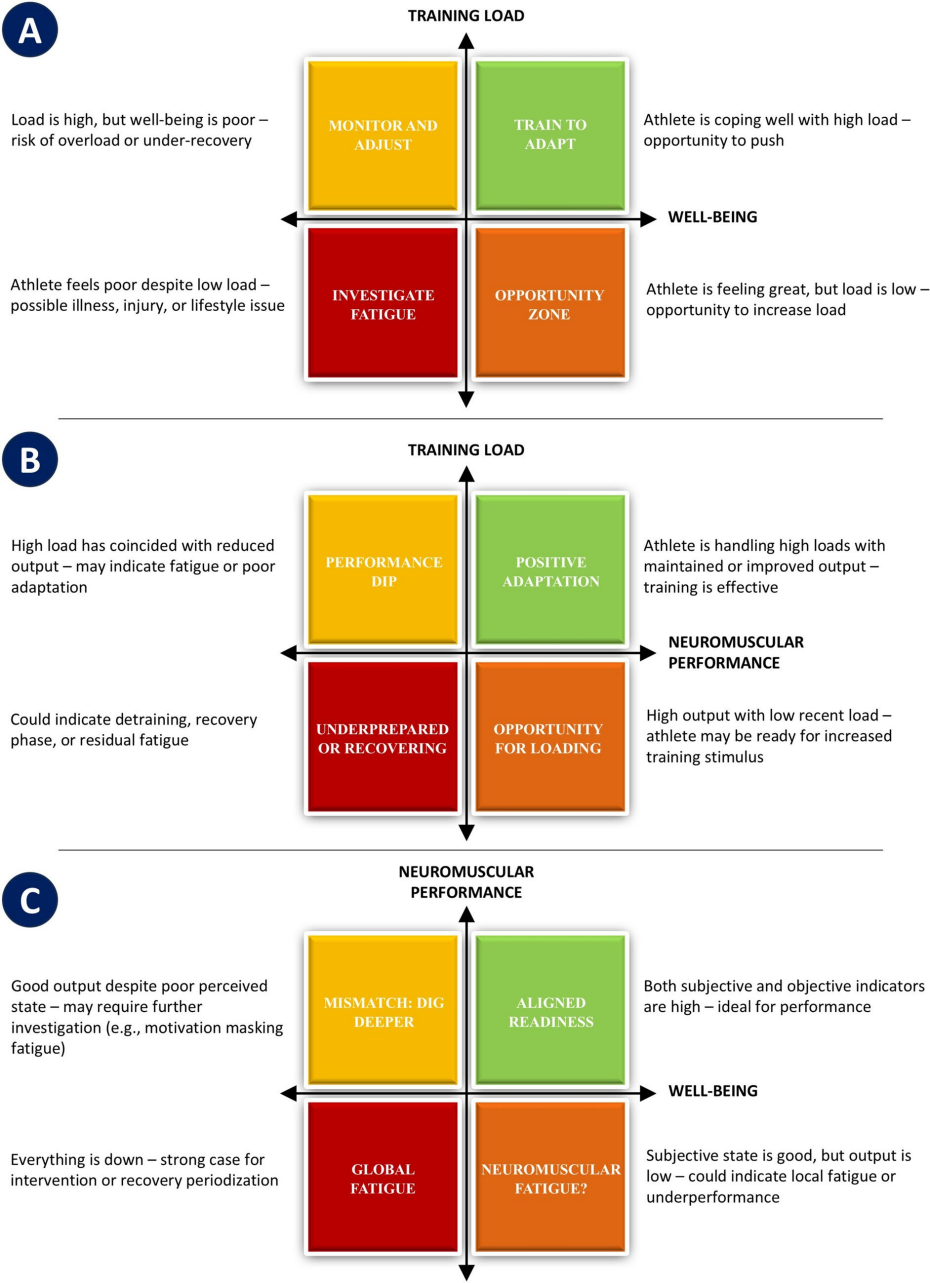
Monitoring quadrants: interactions between training load, neuromuscular performance, and well-being. This figure presents three 2 × 2 quadrants designed to help practitioners interpret key monitoring metrics through a multivariate lens. Each quadrant illustrates a distinct relationship between athlete response variables, allowing for contextualized decision making based on individual profiles. Color-coded zones reflect practical risk and opportunity interpretations (e.g., green for alignment, red for concern), offering coaches a visual framework for adjusting training load and recovery strategies in real time. **A** Training Load × Well-being. **B** Training Load × Neuromuscular Performance. **C** Neuromuscular Performance × Well-being

Thirdly, simplicity should also guide the choice and integration of monitoring tools. Practitioners must critically assess the practical utility of each measurement device or metric by evaluating its ease of use, required resources, reliability, and interpretability. Tools and methods must be practically viable, easily understood by coaching and support staff, and swiftly actionable in day-to-day environments [92]. In this context, ‘invisible monitoring’ has been proposed as an approach to reduce athlete and staff burden by extracting meaningful indicators from data collected during normal training and competition, rather than adding separate testing sessions [99]. However, Leduc and Weaving [99] highlight that adoption requires clearer conceptualization and careful consideration of monitoring burden (frequency and obtrusion), the number of constructs captured by a single tool, and data governance and ethical challenges, including transparent communication with athletes about how data are used and the uncertainty of monitoring-derived inferences. Finally, consistent communication across transdisciplinary teams is essential for effective monitoring [100]. Practitioners may consider adopting visual frameworks, such as quadrant-based approaches, to explicitly separate training load from response and adaptation, and improve the practical linkage between what is measured and what decisions follow (Fig. 3) [101]. These visual aids should integrate individualized SD-based thresholds clearly and succinctly, supporting intuitive, rapid decision making while maintaining robust scientific rigor.

### Interpreting Meaningful Changes: Balancing Error Types

Effective athlete monitoring requires not only the identification of change but also the appropriate interpretation of that change. This process hinges on understanding the balance between Type I and Type II errors, particularly when applying statistical thresholds to real-world decisions in high-performance sport. In individual monitoring, where decisions affect not populations but single athletes, the consequences of these errors differ significantly from traditional group-based analyses. A Type I error (false positive) occurs when a practitioner concludes that a meaningful change has occurred when it has not [95]. In a practical setting, this may lead to unnecessary training modifications, unwarranted concern, or loss of athlete confidence. Conversely, a Type II error (false negative) arises when a real, meaningful change is missed [95]—potentially allowing an at-risk athlete to continue training without adequate recovery or attention, increasing injury risk or performance decrement.

**Fig. 4:**
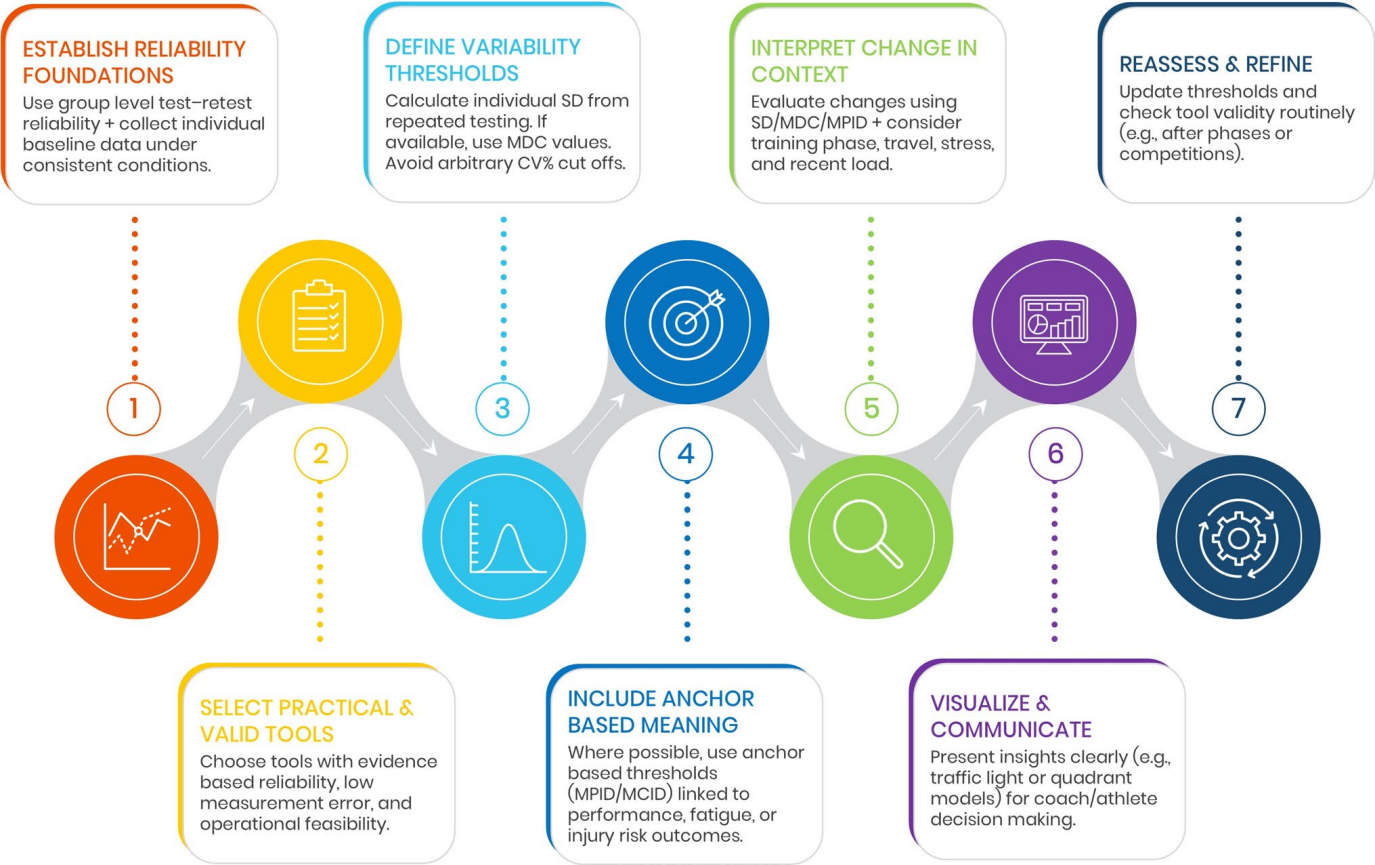
A step‑by‑step applied framework for individualized athlete monitoring. The model integrates group‑level and individual reliability, multiple thresholding methods [standard deviation (SD), minimum detectable change (MDC), minimum practically important difference (MPID)], contextual interpretation, and practitioner communication to support clear and actionable training decisions

Given this context, the tolerance for error must align with the athlete’s performance environment. In high-performance sport, the cost of missing an early sign of maladaptation (Type II error) is often greater than mistakenly identifying a small change that later proves inconsequential (Type I error). Therefore, practitioners may reasonably favour more sensitive thresholds—such as ± 1 SD—when early detection is more important, even at the expense of increased false alarms. In contrast, when clarity and certainty are paramount (e.g., returning to play from injury), more conservative thresholds (e.g., ± 2 SD) may be preferred to avoid premature or unjustified decisions. Importantly, practitioners should interpret changes in the context of the athlete’s typical variability. The use of individual SD-based intervals provides a probabilistic lens through which change can be assessed, but the final judgement must be grounded in the athlete’s clinical and performance history, training context, and recent exposure. For example, a result falling slightly beyond ± 1 SD may not automatically warrant action but could be considered noteworthy if the athlete recently experienced additional stressors such as travel fatigue or competition congestion. In contrast, the same level of deviation might be considered inconsequential during a stable, low-stress training block. This judgement-based process is visually summarized in the implementation flowchart (Fig. 4), which outlines key decision steps from data collection to contextual interpretation. Ultimately, successful interpretation involves embracing uncertainty, not eliminating it. By combining robust individual baseline data, clearly defined thresholds, and practitioner expertise, the monitoring process can achieve both scientific rigor and applied relevance. To further enhance this interpretability, statistical thresholds should be supported by anchor-based methods—linking quantitative change to meaningful outcomes. Thorpe et al. [10] advocate for this approach by highlighting the value of aligning statistical metrics with athlete-centred performance or recovery anchors. As mentioned previously, it was found that a 400-m increase in high-speed running was associated with a 1-unit improvement in perceived fatigue [98], offering a MPID that can inform training decisions. Combining anchor-based MPIDs with distribution-based metrics such as MDC provides a more nuanced lens through which to assess change, ultimately supporting more confident and context-specific action.

## Conclusion

Effective athlete monitoring in elite sport requires a careful balance between scientific rigor and practical feasibility. While conceptual models have progressed toward interpreting training effects rather than relying on binary judgements of readiness, applying these frameworks in real-world settings continues to pose challenges. Practitioners must navigate multiple layers of athlete response, including physiological, neuromuscular, psychological and social factors, while working within resource constraints, managing athlete compliance, and adapting to the complexity of high-performance environments.

This narrative review presents an integrative and pragmatic framework for monitoring training effects, emphasizing the selective use of tools according to their intended purpose, measurement timing, and operational feasibility. It acknowledges both the strengths and the limitations of commonly used monitoring approaches across time scales and domains. Rather than proposing a single ideal solution, this framework encourages a strategic combination of objective and subjective measures, individualized statistical thresholds, and context-aware interpretation to support informed training and recovery decisions.

Readiness-related metrics should not be viewed as definitive outcomes but as operational indicators that provide short-term insight into how athletes are tolerating imposed demands. When interpreted alongside training load, contextual information, and longer-term trends, these indicators can guide timely coaching decisions without losing sight of broader performance objectives. Ultimately, the purpose of athlete monitoring is not solely to detect fatigue or prevent injury, but to support adaptive training processes, promote resilience, and sustain high-level performance over time. By grounding monitoring systems in both scientific evidence and the realities of applied practice, practitioners are better equipped to use data as a decision-support tool that complements, rather than replaces, professional judgement.
